# “Death and Taxes”: Why Financial Compensation for Research Participants is an Economic and Legal Risk

**DOI:** 10.1017/jme.2023.72

**Published:** 2023

**Authors:** Margaret Waltz, Arlene M. Davis, Jill A. Fisher

**Affiliations:** 1. UNIVERSITY OF NORTH CAROLINA, CHAPEL HILL, NC, USA

**Keywords:** Healthy Volunteers, Phase I Trials, Research Participation, Study Payments, Risk, Informed Consent

## Abstract

In the US, research payments are technically taxable income. This article argues that tax liability is a form of possible economic and legal risk of paid research participation. Findings are presented from empirical research on Phase I healthy volunteer trials. The article concludes by discussing the implications of these findings for the informed consent process, as well as for broader ethical issues in whether and how payments for research participation should be regulated.

## Introduction

Although the bioethics literature has a longstanding engagement with questions about the ethics of paid research participation,^1^ the field has been silent about any tax liability associated with these payments. In the US, research payments are technically taxable income. However, study compensation is often relatively nominal or classified even as a *de minimis* benefit, meaning “one for which, considering its value and the frequency with which it is provided, is so small as to make accounting for it unreasonable or impractical.”^2^ Such research payments are simply invisible to the larger income tax system. Yet, when an institution, such as a university or an independent research facility, provides an individual with compensation of $600 or more during a single year, that institution must report the total payment amount directly to the US Internal Revenue Service (IRS) through a Form 1099. Because an individual may be required to pay taxes on any income earned on amounts starting at $400, the 1099 issued provides notice of a potential tax liability for the participant.

While tax liability may be a non-issue for much human subjects research, some clinical trials offer more substantial compensation in exchange for participation. Specifically, Phase I healthy volunteer trials offer participants an average of $3,000, with some trials paying over $10,000.^3^ Phase I trials are those that test the safety and tolerability of investigational drugs as part of the research and development done by the pharmaceutical industry. Healthy volunteers are the preferred participants for these studies because they can help determine the safety profile of a drug without the confounding effect of an underlying disease or condition, and they are easier to recruit for these trials than are affected patients.^4^ Additionally, some have argued that it is more ethical to enroll healthy volunteers in Phase I trials because patients might be particularly prone to a problematic therapeutic misconception when enrolled in a clinical trial that has no measures of drug efficacy.^5^ At the same time, because healthy volunteers have no possibility for direct medical benefit from their participation, they are recruited to these trials through financial incentives.^6^ The payment amounts are typically larger than for other research studies because Phase I trials often require a confinement period during which participants check in to a research clinic for the trial and spend days or weeks in the facility before they can leave.^7^ Thus, Phase I participation conveys not only the risks of testing investigational drugs, but it often requires a significant time commitment from and burden on healthy volunteers.

Exacerbating the tax liability issue in Phase I trials is the trend for many healthy volunteers to be serial research participants. Many enroll in multiple clinical trials per year, and some have a history of enrolling in Phase I trials for decades.^8^ This trend results from social and economic inequalities in the US that motivate healthy volunteers to enroll, making clinical trials particularly appealing to the most disadvantaged segments of the population.^9^ Importantly, a disproportionate number of healthy volunteers are racial and ethnic minorities, and Black men have a longer history of enrolling in Phase I trials and have participated in a greater number of these studies than other groups.^10^ Some healthy volunteers have also been referred to as “professional” participants because they pursue clinical trials as if they are a full-time job and even travel extensively across the US to enroll in new trials.^11^ These patterns of trial enrollment are notable here because serial participation increases individuals’ potential tax liability as payments, and therefore earned income, accumulate over the course of a calendar year.

In this article, we argue that there is an underappreciation of tax liability as a risk of paid research participation. In Phase I trials, the amount owed to the IRS based on income earned from research participation has the potential to be a significant sum, particularly for serial healthy volunteers. Yet, IRBs do not currently require investigators to include this information in consent documents as a form of possible economic and legal risk. To illustrate the importance of this issue, we draw on empirical research with healthy volunteers, focusing on their awareness of tax liability, how tax liability can be a perverse incentive structuring their research participation, and their perceptions of research compensation as taxable income.

The literature has debated whether serial research participation should be considered work,^12^ but from the perspective of the IRS, healthy volunteers are independent contractors who are self-employed and subject to taxes on their research income. Research institutions are well acquainted with their duty to report independent contractor payments to the IRS. As part of the review of human subjects research, institutional review boards (IRBs) facilitate this characterization. They review the study purposes that would necessitate collection of social security numbers from participants, approve the amount and schedule of research payments, and establish the information about payment that must be included in informed consent documents. Indeed, when participants receive financial compensation for their research involvement, consent forms may include language about potential tax liability associated with such payments. Inclusion of this information appears to be quite common in consent forms for Phase I healthy volunteer trials, but the details provided about the potential tax consequences of participation are often minimal or, at times, confusing (Table 1). In this way, consent forms can be read as dispatching with a duty to inform, while also obscuring a participant’s responsibility for determining how their own income taxes may be affected by research payments. And while a consent form may give notice of physical risks or research concerns related to simultaneous or serial enrollment in clinical trials, we have seen no consent form offer notice about the potential impact of such participation on tax liability.

**Table 1:** Examples of Tax Language in Clinical Trial Consent Documents

Compensation may need to be reported to the Internal Revenue Service (IRS) as taxable income.
It is important for you to note that you will need to report this compensation as personal income on your next income tax return.
We ask that you notify the Internal Revenue Service (IRS) of this income. A form 1099-MISC (for miscellaneous income) will be mailed to you for you to report this compensation to the IRS.
By law, we must report compensation totaling over $600 provided to any volunteer in single calendar year to the Internal Revenue Service.
To receive payment, you must provide your social security number, name and address so that we can comply with IRS (Internal Revenue Service) reporting requirements. When payments are reported to the IRS we do not let them know what the payment is for, only that you have been paid. If you do not wish to provide this information you can still take part in this study but you will not be paid.
No deductions for any state or federal withholding or any other similar taxes will be made and you are solely responsible for reporting such payments on your state and federal income tax returns.
You must give your social security number to receive payment. No deductions for state or federal withholdings (or other taxes) will be made on your behalf. You are responsible for reporting study payments on your federal and state tax returns for the payment of taxes. Taking part in this study does not make you an employee of the Sponsor, study site, or the US Food and Drug Administration.
Payment received as compensation for participation in research is considered taxable income to the research study participant. If payment to an individual exceeds $600 in any one calendar year, XXX University is required to report this information to the Internal Revenue Service (IRS). Research study participant payments to a non-employee of XXX University exceeding $600 during any calendar year will result in a 1099 (Miscellaneous Income) form being issued to the individual and a copy sent to the IRS.
Please be advised that compensation for participation in a study is taxable income. Personal information about you, including your name, address, and social security number, may be released for the purpose of payment and for tax reporting to the Internal Revenue Service (IRS). XXX (study site) will issue you an IRS Form 1099-MISC, Miscellaneous Income, listing your compensation as reportable income. Non-resident aliens without a social security number or tax identification number (TIN) may be subject to withholding of 30% and U.S. residents without a social security number will be subject to a withholding of 24% at the time of payment under Internal Revenue Code Section 1441.

Source: Clinicaltrials.Gov. The examples come from US clinical trials with consent documents included in their record that enrolled healthy volunteers in 2019 or later. All examples were included in the section of the consent form on study payments.

In this article, we argue that there is an underappreciation of tax liability as a risk of paid research participation. In Phase I trials, the amount owed to the IRS based on income earned from research participation has the potential to be a significant sum, particularly for serial healthy volunteers. Yet, IRBs do not currently require investigators to include this information in consent documents *as a form of possible economic and legal risk*. To illustrate the importance of this issue, we draw on empirical research with healthy volunteers, focusing on their awareness of tax liability, how tax liability can be a perverse incentive structuring their research participation, and their perceptions of research compensation as taxable income. We conclude by discussing the implications of our findings for the informed consent process, as well as for broader ethical issues in whether and how payments for research participation should be regulated.

## Methods

This article draws upon a mixed-methods, longitudinal study of healthy people who had enrolled in at least one Phase I trial. The purpose of the study was to investigate how healthy volunteers’ perceptions of, decisions about, and behaviors surrounding clinical trial participation change over time. We were particularly interested in perceptions of risks and benefits, but we also were attentive to how enrollment decisions were influenced by factors related to specific clinical trials, along with situational factors relevant to participants’ everyday lives. Participation in our study involved up to five in-depth, semi-structured interviews, as well as data collection about the clinical trials for which participants screened and enrolled while in our study. Detailed descriptions of our methods and instruments have been published elsewhere.^13^

We recruited participants for our study at seven US Phase I clinics to aid in identifying healthy people who were actually enrolled in a clinical trial. Clinic locations were selected so that our sample was drawn equally from the Eastern, Midwestern, and Western regions of the United States. Participants were enrolled in our study from May to December 2013, and each participant was followed for a three-year period. Our study was reviewed and approved by the Biomedical Institutional Review Board at the University of North Carolina at Chapel Hill, and all participants provided written informed consent.

Our sample included 178 participants, of whom 166 (93.3%) were retained for the three years of our study. As with prior studies of healthy volunteers,^14^ our sample was predominantly male and, relative to the US population as a whole, included an overrepresentation of racial and ethnic minorities (Table 2). At the time of enrollment in our study, our participants had a median age of 39. Nearly half of the participants had an annual household income of less than $25,000. A third of our sample held part-time jobs, 41% did not have any formal employment, and only a quarter had full-time work. Study participants also had considerable clinical trial experience: only 21% were participating in their first clinical trial, 28% had participated in 2 to 4 trials, 25% had participated in 5 to 10 trials, and 26% had participated in more than 10 trials.

**Table 2:** Demographics of Study Participants (N=178)

	n	%
**Gender**		
Women	47	26.4%
Men	131	73.6%
Non-Hispanic white	57	32.0%
Black / African American	72	40.4%
American Indian	2	1.1%
Asian	6	3.4%
Hawaiian / Pacific Islander	2	1.1%
More than one race	13	7.3%
Hispanic^1^	38	21.3%
**Born Outside of the U.S.**	35	19.7%
**Age**		
18-21	6	3.4%
22-29	34	19.1%
30-39	58	32.6%
40-49	54	30.3%
50+	26	14.6%
**Household Income** ^2^		
Less than $10,000	30	16.9%
$10,000 to $24,999	52	29.2%
$25,000 to $49,999	71	39.9%
$50,000 to $74,999	13	7.3%
$75,000 to $99,999	7	3.9%
$100,000 or more	4	2.2%
**Educational Attainment**		
Less than high school	12	6.7%
High school or GED	37	20.8%
Some college	52	29.2%
Trade/technical/vocational training	19	10.7%
Associate’s degree	21	11.8%
Bachelor’s degree	32	18.0%
Graduate degree	5	2.8%
**Employment Status** ^3^		
Full-time/Business owner (self-employed)	45	25.3%
Part-time/Independent or Irregular Contractor	60	33.7%
Unemployed/Retired	73	41.0%
**Clinical Trial Experience**		
1 study	38	21.3%
2-4 studies	49	27.5%
5-10 studies	45	25.3%
11-200 studies	46	25.8%

1 The category Hispanic includes all racial groups, of which we have those in our sample who identified as white, Black, more than one race, American Indian, and Native Hawaiian/Pacific Islander.

2 Data for household income was not reported by one participant.

3 These data are based on consolidated definitions of each employment category that we used to standardize self-reported data from participants.

The data for this article are drawn from all five waves of interviews conducted during the study (i.e., at enrollment, 6-months post-enrollment, 1-year post-enrollment, 2-years post-enrollment, and 3-years post-enrollment). A specific question about taxes was not asked during these interviews. Instead, participants brought up the topic on their own accord. All interviews were recorded, transcribed, and coded using Dedoose software. To find excerpts that were relevant to the theme of paying taxes on study compensation, we used Dedoose’s search function to find any discussion of the terms “tax” or “IRS.” We exported these excerpts, further coded them to create subthemes, and analyzed the ethical implications of participants’ experiences and perspectives of the tax issue. When relevant, we also contextualized participants’ comments in their broader history of clinical trial participation and/or other important financial or employment situations. When quoting participants below, we used pseudonyms to protect their confidentiality.

## Results

### Awareness of Tax Liability

Participants in our study had varying levels of awareness and ways of handling their tax liability for Phase I research payments, ranging from not knowing they had to pay taxes on their research payments, knowing they had to pay taxes but struggling to do so, to handling taxes in a savvy, entrepreneurial way. Those who were not aware that they had to pay taxes on their research payments described the “tax-free” nature of trials as a participation benefit. When asked if the opportunity to make money shaped their view of clinical trials, an interviewee named Brett, a Black man in his twenties who had participated in over 10 trials, said, “Oh, yeah, of course. Yeah, always. Especially… when it’s tax-free. It was sick. It was sick.” For Brett and others, the lump-sum payment was perceived as a benefit because it made participation seem more lucrative. As Everett, a Black man in his forties, explained, people “like that free money… They don’t take out the taxes… Like when you do a study for like $5000, they give you a check for $5000. People like seeing that.” Moreover, this belief that research payments were tax free influenced some people’s decision to take part in Phase I trials. This was the case for Bruce, a white man in his forties who had participated in over 20 trials. He said, “The longest study I’ve done is a month at [research clinic], and it paid $9000, so I was like, you know, ‘I’ll do it. What the hell. Tax free.’”

Many interviewees became aware only *after* they had already participated in a study that Phase I participants have to pay taxes. One such interviewee, Amanda, a white woman in her fifties, said she was left shocked when she received a 1099 in the mail after she participated in her first trial. When asked if enrolling in studies gives people a financial advantage over those who do not participate, she responded, “No. Because you have to pay your taxes off of what they give you, yeah… So when you get the check or whatever, they give it to you with no state taxes, no federal taxes, no nothing, nothing taken out.” Thus, at least for Amanda, the financial benefit of enrolling in a clinical trial may be negated by the tax consequences.

Other participants were aware that research payments were taxable income, but they did not know how they could afford to pay their taxes. In fact, Roman, a Black man in his thirties who had completed an estimated 200 trials over two decades, referred to Phase I healthy trial participation as a “dirty business” and likened it to selling drugs. He said, “For every time that you take that money…, it’s technically, right at that moment, tax-free. If you ride [under] the radar, which I did this in the beginning of my study career, doing studies, I did just enough whereas I didn’t have to pay taxes because I was scared to pay money, ‘cause I didn’t know how I was gonna pay it. So, I was like, wow, if they tax me off this tax-free money, so to speak, I may owe them a lot of money, so I’m just gonna ride the rail… That’s when the similarity of the drug trade and this trade go hand and hand because at that point, you’re doing something semi-illegal… You’re not trying to pay your taxes… The drug dealer, he’s out there trying to avoid the police. He’s trying to stay a step ahead of them. I’m trying to stay a step ahead of the IRS.” As Roman illustrates, not knowing how to pay taxes means that some participants avoid paying taxes, exposing them to further economic *and* legal risk.

This lack of awareness or ability to pay income tax on their study compensation had consequences for participants. Self-identifying as a professional participant, Jason, a biracial man in his thirties, had completed nearly 50 trials in the prior 7 years after quitting his sales job, for which he was also an independent contractor, to enroll in trials full time. Jason owed more than $50,000 in income tax, with between $10,000 and $20,000 resulting from his study participation and the rest from his prior job. He wrestled with the consequences of tax debt for himself and other Phase I healthy volunteers, and he described the difficulty of getting back on track after not having paid taxes: “If a person wasn’t on top of their tax situation and didn’t understand it-. I mean, you talk with people that don’t think they have to pay taxes, yeah, that’s going to be a disadvantage the day they find out that they owe Uncle Sam… And that’s kind of how I got into tax trouble at first; because when I was a 1099 employee several years ago [for the sales job], I didn’t do what I needed to do and, you know, follow up and hire a tax professional to do my taxes. So yeah, so a person could get themselves in a similar situation to the situation that I’m in and really not know how to file their taxes the right way or … put money aside to pay their taxes. So that, you know, that could be a disadvantage for a person.”

Steve, a white man in his 40s who had completed over 70 trials, was in a similar situation. He had not filed taxes for many years, which resulted in a large amount of tax debt from his trial participation. He said, “I probably will owe about $35,000 [laughs]. And most of that is because what the IRS sees on all those years is me doing clinical trials where they didn’t take any taxes out. You know, I just got paid in checks without any taxes [withheld], so I’m supposed to pay taxes to the IRS all those years for all those clinical trials.” He described finally dealing with his tax debt “after being kinda off the grid for some twenty-five plus years not filing any taxes with the IRS” because he was pursuing a K-1 fiancée visa for the mother of his daughter to come to the US, which required him being “current with the IRS.” Illustrating the difficulties of getting out of tax debt, he said he owed around $640 for the most recent tax year, but if he paid that, “then I’d be kinda screwed ‘cause I need that money [for living expenses].” Steve wanted to set up a payment plan, but because he was still not up to date on his taxes for prior years, this was not a simple solution. He explained, “But before you can set up a payment plan, you have to be current with all of the previous years… You can’t just make payments on [one year] and then ignore the previous years. You have to get it all settled up front before the IRS will allow you to determine what your monthly payments will be.” Despite how daunting this situation was, Steve had received some positive news from the IRS: “Fortunately, they’re only requiring me to go back to 2001, so you know, 1988 through 2000, I guess they’re letting me slide on those.” Regardless, Steve’s situation was such that even a single-year tax bill of $640 was unaffordable, so the possibility of owing $35,000 for all years combined became a catastrophic obstacle to bringing his fiancée and daughter to live with him in the US.

Other participants who knew that they had to file taxes put money aside throughout the year to be able to handle their tax liability. These participants referred to setting money aside as the “smart” thing to do, with some judgement of those who did not do so. For instance, AJ, a Black man in his twenties, said, “I always just put money aside, you know, for … taxes and whatnot… ‘Cause at the end of the year, you know, they come at you with like a nice like $2,000 something,… what you gotta pay back. But if you’re not smart-, if you’re not smart like me-. You put some money aside, you know, here and there, and then you just, boom, pay ‘em. They [the IRS] don’t care, you know.”

While saving money for their taxes allows participants to manage their tax liability, it is not always feasible for people to do so, especially for those who are unbanked. This was the case for Marco, a Hispanic/biracial man in his twenties. He regularly used predatory check cashing stores when he received payments of any kind. His first clinical trial paid him $7,600, which literally became cash for him to carry around and spend. Marco described how he did not spend this money “wisely,” saying, “it was a long time since I had a lot of money like that, you know, at one time… So I’ve seen how it could get to you. And I’ve actually seen how it could get to you to have-. Somebody [in an apartment] down the hall, [his] father won the lottery… And I know him, and I see how … it could help you or ruin your life. And in one year, I see how you can go broke … and then end up in jail for not filing certain taxes.”

While Marco revealed the potential consequence of not having money to pay income taxes, many interviewees who did set money aside seemingly ignored or were oblivious to the fact that doing so requires the financial ability to save. Ron, a Black man in his forties, said, “You really have to be on top of your money because you’re not taking out money regularly to pay taxes. So you get hit with tax, you know, when it comes around to tax time. But for me, I’m a ‘live within my means’ kind of person, so it’s fine… And on April 16^th^, I’m sitting back and resting comfortably.” Similarly, Helen, a white woman in her thirties, described the dangers of not putting money aside throughout the year. She said, “My husband makes sure that I sit down and we take out the money for this and set it aside to pay the taxes with. And I know these young folks don’t do that… And they’re gonna be in a world of trouble ‘cause they’re not going to have that money set aside to pay it. You know, but when you get those I-9s [sic], you have to pay taxes on this money, and they just act like it’s footloose and fancy free, y’know, but it’s not.” While both Ron and Helen experienced financial precarity that motivated their clinical trial participation, both were highly educated. Helen had received a bachelor’s degree and was pursuing a nursing degree, and Ron had a graduate degree.

Other participants who were aware of needing to pay taxes from research payments went beyond saving money throughout the year and found savvy ways to reduce their tax liability, such as through writing off expenses. For example, Henry, a white man in his fifties, said, “The study income is 1099 income, so 1099 income is much more beneficial from a tax standpoint because you can write off all your expenses and so forth… So, you know, the nice benefit, like I say, of study money is you definitely have expenses that can offset pretty much all of the income that you get.” Some participants were scrupulous about tracking legitimate expenses that occurred for participating, including screening. Yet others, like Henry, were highly strategic about leveraging their expenses when deciding when and where to enroll in trials. He described his choice to enroll in a clinical trial in Florida in order to write off the expenses associated with a personal trip there to see his son play in a golf tournament. This ability to write off expenses meant that, for Henry, the study payment is “all pure profit.”

Much like the knowledge that research payments are subject to income taxes in the first place, such savviness needed to be learned, often from other Phase I healthy volunteers. For instance, after avoiding the IRS because he could not afford to pay taxes, Roman learned that a fellow healthy volunteer got a tax refund instead of owing taxes every year. He described two other people involved in that conversation, saying that one ended up owing money instead of getting a refund and the other never tried to get money back. Roman said that “a couple of years later, the guy that owed the taxes, I bumped into him. I said, ‘Hey, did you ever fix the tax thing?’ He said…, ‘I don’t deal with that no more. I just do the studies. I get my money, and if I owe, I owe; if I don’t, I don’t.’ I said, ‘Well, wow, that’s interesting … because I get taxes back. I itemize everything that I buy for the studies. I’m basically my own boss… Everything the guy told me was true.’” Learning how to be savvy with his tax liability seemingly led Roman to judge healthy volunteers who did not adopt similar accounting methods. He continued, saying, “So you figure he told three people, one didn’t follow through with it at all. One followed through with it but not fully committed and failed. I followed through, committed, and I prospered. So you have people like that, you know, they don’t have the motivation or the understanding to follow through.” Similarly, other interviewees judged those who lacked savviness and tried to avoid paying the IRS altogether. Peyton, a Black man in his forties, said that he looked at healthy volunteers who try to avoid the IRS “like, ‘Yo, you know they’re going to catch you, right, dum-dum?’… And I’ll tell you, one girl they did her like that [the IRS garnished her study compensation]… She was crying, and … I was like, ‘You don’t need to cry. You knew you had to pay that shit.’ Excuse my French… That’s the way it works… I guarantee you they [the IRS] will catch up with you… And I’m doing the right thing… while you running around trying to play it slick. I don’t get it. I just don’t get it. I don’t get it.”

Once the savviness was learned, participants’ ability to navigate or circumvent tax liability was often linked to their identity as entrepreneurs. Rob, a Native Hawaiian man in his forties, said that “if you do this [participate in Phase I trials] full time, you actually get a good sense of business. Like, you know, what goes in, what you got to pay for taxes, and you know, you have to save receipts… Food, lodging, hostel, shelter, motel … wherever you stay, yeah. You can write it all off. I mean, that’s all business expense, you know.” Another interviewee, Bree, a Black woman in her thirties, said participating in Phase I trials “is an entrepreneurial thing because you do have to keep up… [with] your taxes, your deductions. It puts you in the mind of a businessperson ‘cause that’s what you are. So there’s an entrepreneurial thing, and that’s not found [it’s in-born].” Elias, a Hispanic man in his thirties, described getting for tax purposes a business license for being a Phase I participant because “basically, that’s what I am. I’m a contractor. I’m a contractor of my own body to do these studies and everything like that. And then, of course, you know, you pay for all the expenses, like … your travel expenses, you know, renting of the cars, how many miles that you go back and forth, you know, food-wise, clothes that we buy, our phone… Everything’s tax deductible, but it could be, it’s like, oh, God. It’s a pain in the rear at times, but it’s worth it [laughs].” This approach, however, is not necessarily viable for a lot of participants, especially those who participate only in local trials and, therefore, do not accrue many expenses from travel. There is also a worry that this learned savviness might backfire, in that participants begin to take deductions where no legitimate deductions exist.

### Perverse Incentives Around Making Money

Regardless of whether interviewees were savvy or able to pay their income tax bill at the end of the year, the tax liability from research payments resulted in perverse incentives around making money generally as well as specifically in Phase I trials. Depending on the participant, this led to either curbing their trial enrollment or motivating continued enrollment. For instance, many volunteers were incentivized to stay below a certain total annual income threshold in order not to owe taxes. This included savvy interviewees, like Celeste, a Black woman in her twenties. She said, “I [know] a guy, he said he made too much money [in studies], and he wound up owing taxes even with a child [to claim as a dependent]. See, I never made no more than thirty thousand [total income per year]. You understand what I’m saying? So I made just about enough, in other words, to get money back, yeah.”

The incentive to stay below a certain annual income extended beyond a focus solely on tax liability, as too much income jeopardized some interviewees’ ability to qualify for federal or state entitlement programs, as well as to receive other forms of financial aid. When asked what financial disadvantages come with study participation, Victor, a Black man in his forties, described how earning too much money in trials threatened his eligibility for college financial aid. He said, “You do have to pay taxes later on. You have to factor that part into the amount of study [money] that you make. I guess now it’s not as bad as it was before, ‘cause when I was going to school, doing studies affected my financial aid, so the more money I made, the less financial aid I get… It’s like you made as much money as possible without having it affect my financial aid, ‘cause my financial aid is very important to me.”

Other participants cited issues with taxes stemming from receiving too much income from full- or part-time work outside of clinical trial participation. Tess, a white woman in her fifties who had a full-time job, described how the risk of owing taxes in the future outweighs the benefits of having money in the moment. She said, “I probably may not do another study for the rest of the year… Just because my income grows quite a bit. You know, now it’s another $6,000 on top of my income [from my full-time job] … that, you know, is untaxed [from this study]… So now I’m going, ‘Damn, I’m going to have to pay money at the end of the year, if not break even [and not get a refund check].’ So now I’m kind of like, you know, it’s great to have money in my pocket at the moment, but when reality comes around and next year, you know?… If it’s a couple thousand or $1,500 [for another study], I can deal with that, but if I do a couple more two or three thousand dollar studies, I’m going to owe lots of money.” Similarly, Kent, a white man in his sixties who was receiving Social Security payments, said that “if it wasn’t for [my part-time job], it’d be much more likely that I’d be more incentivized [to participate in more studies], but that job-. I’m within, I think, within a thousand or so of going over that minimum-, or that, yeah, that limit. And every dollar I go over that limit, I gotta pay back [to Social Security] next year when I file taxes, so.”

While some interviewees limited their participation in Phase I trials to keep their incomes low enough to not owe taxes, other interviewees were incentivized to continue to participate in trials *specifically* to be able to pay the taxes they already owed. This is not to say that they would not have enrolled in more clinical trials otherwise, but part of their decision making, in terms of timing as well as amount of trial compensation, was influenced by an impending tax bill. Lee, a Black man in his fifties, noted how pharmaceutical companies themselves do not realize the burden of tax liability, saying, “Some people that have put in years and the first thing these pharmaceutical companies might say is, ‘Well, look at the money you’ve made over the years,’ but it’s not tax-free… And so we’re still-, that’s why we got to do another study, so we can pay taxes [laughs].” Lee might not mean it literally when he says that taxes are the cause of his serial trial enrollment as he jokes about the tax implications, but other participants may more acutely feel the connection between their trial participation and their tax debt. With more than $50,000 in tax debt and penalties from not filing taxes on his previous earnings, Jason was quite explicit about how the money he owed to the IRS has kept him involved in clinical trials despite his mixed emotions about enrolling. When asked how long he plans to continue to participate in trials, Jason replied, “I can’t put a, you know, amount of years on it, but … I need to fix my tax situation… So I need to get out from under that tax debt.” The IRS levied Jason’s bank account because of this tax debt, and he noted that he could not hire a tax attorney to help reach a settlement because he could not afford the legal fees. While he wanted to get a stable and well-paying job outside of clinical trials, Jason said, “There’s really not a whole lot of incentive to, you know, get this high-paying job now if that’s gonna make me, that’s gonna force me to pay back more of the taxes that I owe… So it would be more ideal for me to settle my taxes now while I’m not, you know, making the type of money that I envision myself making eventually.” Because of the threat of getting wages garnished, he recounted that it was not “feasible” for him to stop participating in trials until his tax burden was lifted. Yet, it is important to point out that continuing to enroll also exacerbated the situation because he was still earning income for which he would owe taxes.

### Ramifications of Tax Liability for Phase I Trials

Regardless of whether and how tax *liability* influenced participants’ decisions to enroll in clinical trials, one ramification of not withholding taxes from research payments is that the monetary incentives to participate in Phase I trials appeared to be more profitable and reflective of the time commitment than they really were. This raises the question of whether financial incentives unfairly take advantage of healthy volunteers’ misunderstanding of the tax liability associated with their trial involvement. As previously noted, the perception, as well as valorization of receiving a lump-sum (i.e., substantial) payment, permeated participants’ perceptions of Phase I trials. Yet, for those who focused on the fact that a tax bill would follow, they complained that the study compensation was not only not lucrative but also inadequate. For example, Mindy, a white woman in her fifties, noted that “at the end of the year when you have to pay taxes on it, it’s really, you know, I think you’re probably making like 3 bucks an hour to do a trial, yeah.” Travis, a Black man in his forties, similarly described how much work goes into that little amount of money, making the effort seem less worthwhile. He said, “After, you know, minus the taxes, the time, the travel, the this, the that, you know, how many screenings you have to go to and all, you know, it just, at the end of the day, it just-, you know, it was a lot less than what you actually thought you were making.”

This misleading nature of financial compensation earned raises ethical issues around participants’ decision making when they weigh the risks and benefits of Phase I trials without really having a complete picture, including of the economic and legal risks. Lee described the informed consent processes for trials, with people focusing mainly on the physical risks of participation. He said, “The first things that pop out though, people know when you say life and death, but they don’t know when you say death and taxes. They just want the money and they’re, ‘Oh, I got to pay taxes on [it]?” Rufus, a Hispanic man in his fifties, described his own risk-benefit calculation, saying, “The benefits were only monetary and to [fulfill] my curiosity to see what it’s like because I wanted to find out ‘what are the studies and what do they do.’ But I always think that there is a [physical] risk, and I’d rather not run that risk. And you think that you’re going to have that monetary benefit, and there isn’t [actually much] of any, there isn’t like I thought there would be, so I’d say no [to doing it again]. Why take money when the next year you have to return it [in taxes]” (translated from Spanish). Rufus also questioned who or what was ultimately benefiting from healthy volunteers’ participation in Phase I trials. Firm in his stance of not wanting to participate anymore, he explained, “because I really did not find it fair that at the moment of reporting taxes, the government takes much of it away. That stuck in my mind. What is the advantage, I thought? … I mean, who is benefiting the most, is it the participant or the government? So who or what benefits here? That’s the doubt that I have” (translated from Spanish). The unfairness of tax liability from research participation would potentially be more acutely felt by someone like Rufus who had migrated to the US from Central America and had personally and vicariously experienced discrimination related to his legal status in the country.

Because of this financial bait and switch, some participants questioned the fairness of having to pay any taxes on research earnings. Sylvester, a Black man in his twenties, reflected on this unfairness in terms of the risk of physical harm from participation, particularly if one actually suffers a longer-term injury. He said, “I don’t think we should have to pay taxes on this money…. Because you shouldn’t have to pay taxes for a thing like this because we’re doing nothing but service to the people. And nothing bad comes out of this, it’s a hundred percent all good. Only bad that’s coming out of it is if something happens to you in a study to you, your own person, and then you have to deal with [that]. Then, you didn’t get much from it but that one check, and then you still have to pay taxes on it. It’s just weird to me; like, it’s the times haven’t caught up yet. That’s how I feel.” Similarly, Martin, a biracial man in his twenties who believed the government benefits from Phase I healthy volunteers, said that participants “need some type of tax write-off. We need some type of-, ‘cause we’re saving lives… [and are] getting underpaid. And these coordinators and doctors are getting all the money. And the sponsors, too, then the companies are making hella money. Without us, it’s not gonna go.” Others also saw a problem in how much profit the pharmaceutical industry makes in comparison to participants’ research earnings after taxes. Lee said, “We have to pay taxes on x amount of dollars when you get in here. And that’s only right. But … we got the big pharmaceutical companies now making gazillions of dollars off of research alone.” As a result, he said that pharmaceutical companies should give more to healthy volunteers, whether in the form of stocks or just “fair compensation” that factors in that taxes must be paid.

## Discussion

Despite current informed consent processes, it is news to some individuals who enroll in research and receive payments to learn that they are engaged, for tax purposes, in self-employment. From the accounts above, we see a variety of participant responses to their independent contractor status — some surprised by tax consequences, some strategizing around tax liability, and still others hoping to avoid the IRS spotlight altogether. We have described the tax liability that may come, in particular, with Phase I trial participation as well as the potential impact of this liability for healthy volunteers. To further understand the economic and legal risks related to tax liability, it is helpful, then, to turn to fellow independent contractors in other areas of work.

Neglecting financial considerations and glossing over payment structures stem from and contribute to an underappreciation of participant vulnerability.

With the rise of the gig economy through platform companies like Uber, DoorDash, and Airbnb, among so many others, healthy volunteers can now more legibly be seen as another type of independent contractor typically classified as gig workers.^15^ This framework for understanding research participation as an economic activity is helpful because scholars have considered both the ways that gig work is exploitative^16^ and the importance of tax consequences for these workers.^17^ For example, Kathleen DeLaney Thomas has written about the difficulty for low-income independent contractors to budget for and pay taxes, including by tracking expenses that can offset their tax liability.^18^ Thomas states, “gig workers may have a particularly difficult time dealing with their tax obligations because they tend to be inexperienced, are potentially illiquid, and often do not understand the tax rules that apply to them.”^19^ Gig workers who are unfamiliar with paying self-employment taxes may be further saddled with fines and interest on late tax payments. And while taxpayers can work directly with the IRS to arrange payments, tax professionals, such as certified public accountants (CPAs) or attorneys, can often negotiate with the IRS to their client’s advantage. However, retaining professional services creates another expense. At the same time, ignoring their tax liability is not a long-term solution for gig workers. Depending on the circumstances, both civil and criminal penalties may be incurred by taxpayers. Or, to put the point differently, tax liability is a potential form of both economic and legal risk.

Framing healthy volunteers as gig workers also aligns with some participants’ own identities as entrepreneurs.^20^ For these participants, the gross, untaxed 1099 income serves as the incentive to enroll. Because they understand how to leverage their expenses as tax deductions, entrepreneurial participants see clinical trials as more financially beneficial than income they could receive elsewhere and from which taxes are withheld. The savviness of the entrepreneurial participants represents the best-case scenario when it comes to minimizing one’s tax liability from Phase I research enrollment. However, these participants must have legitimate expenses that offset their tax liability in the first place. These expenses for travel, lodging, and food ultimately take away from their clinical trial earnings, even if they minimize how much is owed to the IRS. And if an individual decides that trial participation is their full-time employment, they may fall within a tax category advising quarterly payments and careful scrutiny of any office, vehicle, phone, or wardrobe they characterize as necessary business expenses. In this way, entrepreneurial participants who navigate these tax-related hurdles are merely doing better than their less savvy counterparts who are devoting similar resources in order to enroll in trials but paying taxes on their total earnings.

Outside of the realm of savvy Phase I healthy volunteers, there are participants who do not realize that they are independent contractors who must pay income taxes on their study earnings, much like Thomas describes for gig workers more generally.^21^ This lack of understanding illustrates that, even with institutional attention to literacy levels for consent processes and forms,^22^ investigators are not offering information about tax liability in a way that is clear, or at all, begging the question of whether the terms of agreement are fair for participants. Such inattention to financial concerns contrasts sharply with the amount of information provided about physical risks and other study burdens that are, at least in theory, typically emphasized to prospective healthy volunteers to support their autonomous decision making about study participation. Consent forms and processes are not doing their intended work when they neglect to offer more complete risk information. Perhaps past preoccupations with research payments — chiding researchers for portraying payments as a benefit and overwrought concerns about payment amounts luring individuals into risky studies — have resulted in a glaring omission: avoidance of any consideration of how payments *should* inform decision making about research participation.

Neglecting financial considerations and glossing over payment structures stem from and contribute to an underappreciation of participant vulnerability. Saving for future income tax payments may not be possible for individuals who are barely making ends meet and use clinical trial participation as a financial safety net.^23^ Additionally, healthy volunteers may be unbanked, which makes saving money for future IRS payments, or any transactions with the IRS, all the more difficult. Setting aside funds also assumes that participants can anticipate how much income tax they will owe, yet the US tax system is notoriously opaque and unpredictable.^24^

While some healthy volunteers may avoid tax liability by not reporting their research earnings to the IRS, failing to report income is a risky decision that not only raises serious legal consequences but other economic and social disadvantages. These disadvantages include barriers to and difficulties getting financial aid, as seen with Victor. They also extend to other events where tax forms may need to be submitted, like applying for subsidized housing or qualifying for a fiancé(e) visa, as seen with Steve. Importantly, the people who need such services are forced to deal with their back taxes and liability, unable to evade the IRS like others who are not (presently) interacting with the various services that require being current on taxes. Despite the substantial amount of taxes that can accrue, even bankruptcy may not be an option since taxes need to be filed on time to meet the requirements to discharge the debt and legal assistance is often required.^25^

Another economic risk of paid research participation stems from the way that individuals’ annual income may affect their eligibility for an array of government and other services. This vulnerability can clearly be seen in healthy volunteers’ perverse incentives to stay below a certain annual income level or base decisions to participate on their estimated tax liability from previous studies. Crucially, participants may be unaware of these negative financial consequences from their research involvement until they actually find themselves in these adverse situations. As a result, those who are already vulnerable are left potentially more vulnerable through these cumulative disadvantages, an important point of consideration as participant payments have traditionally only been scrutinized for their potential “coercive” impact^26^ and not for how they otherwise raise additional vulnerability concerns.^27^

Because of these potential harms to study participants, IRBs should recognize tax liability as an economic and legal risk. By classifying tax liability as a study risk that requires IRB oversight, investigators will then have to manage that risk, which has important implications for the review of the informed consent process for studies like Phase I trials and others that offer more substantial payments. In particular, IRBs should require investigators to address the possible economic and legal risks associated with all study payments. Informed consent forms should clearly notify participants that research earnings may be taxable income, and investigators should discuss potential tax liability along with other risks as part of the consent process. This is not to suggest that research participants should be given legal advice on their tax liability, but to meet the ethical requirement of respect for persons, there is a need for clear communication about these risks. A potential participant deserves to know the possible financial and legal impact of study payments related to their participation in order to make an informed decision about study enrollment. To discuss other kinds of risk and omit these risks appears disingenuous and characterizes the payment as something it is not.

The current approach to informing participants about tax consequences of their research participation is insufficient given the extent to which healthy volunteers in our study were unaware and generally uninformed about the tax implications of their study earnings. Therefore, informed consent forms and processes for studies with payments over $600 should include language such as:

By accepting payment for your participation in this research study, the Internal Revenue Service (IRS) classifies you as an independent contractor. This means the IRS sees you as “self-employed” and that you may owe income tax on a percentage of your payment. No tax is being withheld from the payment you receive, but we will report to the IRS the payment we make to you using a 1099 Form. You are responsible for filing your taxes and paying the IRS any income tax you owe on your study compensation, even if you are not a US citizen. Total taxes owed are based on your annual income, which could include payments from other research studies. More information about managing your income taxes and any deductions that may lower the amount of taxes you owe can be found at *www.irs.gov/businesses/small-businesses-self-employed/manage-taxes-for-your-gig-work*.

In addition to changes to consent forms and processes, tax liability should be factored into discussions of research incentives more broadly. Historically, ethicists and IRBs have been primarily concerned with the incentive amount provided to participants due to fears of undue influence.^28^ However, given the gap between concern for undue influence and no concern for tax liability, it is not just the participants who are currently misappreciating what incentives look like. By not taking the amount of taxes owed on research earnings into account, discussions of the gross payment and whether participants are being paid *too much* ignore the fact that participants often owe the IRS a share of those earnings. Beyond the amount participants actually earn in study compensation after taxes, the unrealistic burden on participants of saving some of the money to use when they file their taxes ignores the role that the research enterprise plays in exacerbating participants’ social, economic, and legal vulnerabilities. And in the context of gig work, placing this burden on economically vulnerable research participants to save money and manage their independent contractor status can be seen as an unfair at best and exploitative at worst.^29^

## Conclusion

While changes to consent forms and discussions of incentives can help manage the risk of tax liability, there are broader mechanisms that could be used to address this risk. Importantly, none of the healthy volunteers in this study said that income tax should be withheld from their payments. For them, the gross payment is a key part of the incentive to participate. That finding should give us pause given research payments currently come without any information for participants about what their after-tax, net payment might be. And even those participants with some understanding of the financial risk view the gross payment as a benefit. While it may not be what participants prefer, there is an ethical argument to be made for withholding taxes from research payments.

However, there is a question that the participants in our study raised about whether research compensation should be taxable income at all. Unlike prize or lottery winnings categorized under the tax regulations pertaining to gambling, participants take on potential physical risk in their contributions to science and/or drug development, which has a broader social value. Unlike other organizations utilizing gig workers, research facilities are not skirting labor laws to hire workers as independent contractors to keep labor costs low.^30^ Research participants are clearly not employees of research clinics even if they are engaged in clinical labor.^31^ Therefore, research compensation should be categorized as non-taxable income. Until that time, the research oversight system must ensure that detailed information about tax liability is provided to participants so they are better equipped to consider enrollment and manage the potential economic and legal risks that might follow from their trial participation.
